# Overexpressing CsSABP2 enhances tolerance to Huanglongbing and citrus canker in *C. sinensis*

**DOI:** 10.3389/fpls.2024.1472155

**Published:** 2024-10-08

**Authors:** Liting Dong, Shuang Chen, Lanyue Shang, Meixia Du, Kaiqin Mo, Shuwei Pang, Lin Zheng, Lanzhen Xu, Tiangang Lei, Yongrui He, Xiuping Zou

**Affiliations:** Citrus Research Institute, Southwest University/National Citrus Engineering Research Center, Chongqing, China

**Keywords:** Huanglongbing, citrus canker, salicylic acid, methyl salicylate, salicylic acid binding protein 2, resistance

## Abstract

Huanglongbing (HLB) and citrus canker, arising from *Candidatus* Liberibacter asiaticus (*Ca*Las) and *Xanthomonas citri* pv. *Citri* (*Xcc*), respectively, have been imposing tremendous losses to the global citrus industry. Systemic acquired resistance (SAR) has been shown to be crucial for priming defense against pathogen in citrus. Salicylic acid (SA) binding protein 2 (SABP2), which is responsible for converting methyl salicylate (MeSA) to SA, is essential for full SAR establishment. Here, we characterized the functions of four citrus SABP2 genes (*CsSABP2-1*, *CsSABP2-1^V18A^*, *CsSABP2-2* and *CsSABP2-3*) against HLB and citrus canker. *In vitro* enzymatic assay revealed that all four proteins had MeSA esterase activities, and CsSABP2-1 and CsSABP2-1^V18A^ has the strongest activity. Their activities were inhibited by SA except for CsSABP2-1^V18A^. Four genes controlled by a strong promoter 35S were induced into Wanjincheng orange (*Citrus sinensis* Osbeck) to generate transgenic plants overexpressing *CsSABP2*. Overexpressing *CsSABP2* increased SA and MeSA content and *CsSABP2-1^V18A^* had the strongest action on SA. Resistance evaluation demonstrated that only *CsSABP2-1^V18A^* had significantly enhanced tolerance to HLB, although all four *CsSABP2s* had increased tolerance to citrus canker. The data suggested the amino acid Val-18 in the active site of CsSABP2 plays a key role in protein function. Our study emphasized that balancing the levels of SA and MeSA is crucial for regulating SAR and conferring broad-spectrum resistance to HLB and citrus canker. This finding offers valuable insights for enhancing resistance through SAR engineering.

## Introduction

Huanglongbing (HLB), leads to significant economic losses in the global citrus industry, with estimates reaching billions of dollars (Wang, 2019; Singerman and Rogers, 2020). HLB is caused by a phloem-restricted gram-negative bacterium belonging to the genus “*Candidatus* Liberibacter” which comprises three species: “*Candidatus* Liberibacter asiaticus (*Ca*Las),” “*Candidatus* Liberibacter africanus (*Ca*Laf)” and “*Candidatus* Liberibacter americanus (*Ca*Lam)” (Bové and Barros, 2006; Wang and Trivedi, 2013). *Ca*Las is the most prevalent pathogen in the world. Additionally, citrus canker, which results from *Xanthomonas citri* subsp. *citri* (*Xcc*), is another serious bacterial disease (Shahbaz et al., 2023). Almost all commercial citrus cultivars are at risk of infection from HLB and canker (Vojnov et al., 2010; Xu et al., 2023). Currently, chemical approaches play a primary role in controlling the spread of HLB and canker. However, chemical pesticides may cause some adverse effect on natural environment, on human health, as well as on other organisms. Breeding for resistance is the preferred strategy for managing HLB and citrus canker. Genetic engineering techniques have been employed to improve resistance against these diseases (Conti et al., 2021). Developing broad-spectrum resistance genes is an effective approach for enhancing dual resistance to both HLB and citrus canker (Hao et al., 2016; Xu et al., 2023).

Systemic acquired resistance (SAR) triggered by the initial infection of a pathogen in local leaves, provides prolonged and broad-spectrum protection throughout the entire plant (Fu and Dong, 2013). When pathogen invades plant, mobile SAR signals generate in primary (local) infected tissue and then spread through the phloem to establish SAR in secondary (distal) uninfected tissue, which ultimately activates enhanced defense responses against pathogen infection (Fu and Dong, 2013; Vlot et al., 2021). SAR is characterized by non-host resistance and no specificity in the pathogen, enabling it to combat a wide variety of pathogens (Spoel and Dong, 2012). Salicylic acid (SA) is a central signal for SAR establishment (Kim and Lim, 2023). Methyl salicylate (MeSA) serves as a mobile signal of SAR, which can transport SAR signals through the phloem (Park et al., 2007). At the infected cells, SA accumulates and is converted into MeSA by salicylic acid carboxyl methyltransferase (SAMT). MeSA then travels via the phloem to uninfected distal tissues, where it is converted back to SA by salicylic acid binding protein 2 (SABP2), resulting in the activation of SAR (Du and Klessig, 1997; Seskar et al., 1998; Park et al., 2007). MeSA also mediates plant-plant defensive communication through diffusing to the air (Gong et al., 2023). Moreover, plant pattern-triggered immunity (PTI) and effector-triggered immunity (ETI) trigger the induction of SAR and in turn SAR can enhance PTI and ETI response, which ultimately improves the overall resistance of plants (Fu and Dong, 2013). It has been shown that engineering key genes involved in SAR can confer crops with broad-spectrum disease resistance (Zhao et al., 2024).

SAR-mediated resistance has been applied in creating disease-resistant citrus varieties. NPR1 (*Non-expressor* of *pathogenesis-related genes 1*) serves as a key mediator for SA-regulated SAR (Backer et al., 2019). The overexpression of *AtNPR1* from *Arabidopsis thaliana* in citrus has increased resistance to both HLB and citrus canker (Zhang et al., 2010; Dutt et al., 2015; Boscariol-Camargo et al., 2016). Overexpressing a NPR1 homolog, CtNH1, from *Citrus maxima* increases resistance to citrus canker in Hamlin sweet orange (*C. sinensis*) (Chen et al., 2013) while overexpressing *CsNPR1* from Sweet orange confers enhanced tolerance to HLB in Shatian pomelo (*Citrus grandis* (L.) Osbeck) (Wu et al., 2021). We previously showed that the overexpression of a citrus NPR1-like (*CiNPR4*) gene in Wanjincheng orange (*Citrus sinensis Osbeck*) enhanced tolerance to HLB (Peng et al., 2021). Then, we also demonstrated that overexpressing the citrus SAMT homology *CsSAMT1* enhanced tolerance to HLB through increasing SA and MeSA levels in Wanjincheng orange (Zou et al., 2021). Moreover, this increased MeSA as a community immune signal in transgenic plants can confer primed tolerance to HLB in neighboring trees (Chen et al., 2013). Additionally, Soil-applied SAR inducers, such as Imidacloprid, Thiamethoxam, and Acibenzolar-S-Methyl reduces incidence of citrus canker (Graham and Myers, 2011); MeSA signaling enables the plant SAR to respond effectively to *Xcc* attacks (Nascimento et al., 2022). Collectively, these findings suggest that engineering SAR-mediated resistance is a valuable strategy for improving disease resistance in citrus.

SABP2, characterized as a methyl salicylate esterase, is essential for successful establishment of SAR in some species including Arabidopsis, tobacco, poplar and bean (Kumar and Klessig, 2003; Park et al., 2007; Tripathi et al., 2010; Li et al., 2018; Xue et al., 2021). Recently, the overexpression of *NtSABP2* from tobacco has been shown to enhance tolerance to HLB in transgenic ‘Hamlin’ sweet oranges (Soares et al., 2022). However, the roles of citrus SABP2 in regulating SAR against pathogen infection is not understood well. Our previous study showed that four citrus *SABP2* genes *CsSABP2-1*, *CsSABP2-1^V18A^*, *CsSABP2-2* and *CsSABP2-3* had potential role in response to *Ca*Las infection (Zhao et al., 2021). Here, we further investigated their functions in resistance to HLB and citrus canker. *CsSABP2-1^V18A^* was an artificial mutant of *CsSABP2-1* that Val-18 was mutated to Ala-18. Biochemical characteristics of the CsSABP2 proteins were evaluated using recombinant CsSABP2 expressed in yeast. Subsequently, the roles of four *CsSABP2* genes in conferring tolerance to HLB and citrus canker were examined by overexpressing *CsSABP2* in Wanjincheng oranges, which are susceptible to HLB and citrus canker.

## Materials and methods

### Plant and bacteria materials and growth conditions

All Wanjincheng orange (*Citrus sinensis* Osbeck) plants utilized in this research were maintained in a greenhouse at the National Citrus Germplasm Repository, located in Beibei, Chongqing, China. Citrus materials carrying *Ca*Las were collected from infected orchards in Guangxi and *Ca*Las is proliferated by grafting the infected branches on heathy Wanjincheng orange seedlings in greenhouse. The *Xcc* strain *Xcc* YN1 employed by this study were cultured and prepared as described by Peng et al. (2017).

### Vector construction

The coding sequences of *CsSABP2-1*, *CsSABP2-2* and *CsSABP2-3* were amplified from Wanjincheng orange cDNA using the primers CsSABP2-1-f/SABP2-1-r, CsSABP2-2-f/SABP2-2-r and CsSABP2-3-f/SABP2-3-r, respectively (**Supplementary Table S2**), and cloned into the pGEM-T Easy vector (Promega, Madison, WI, USA) to produce pGE-SABP2-1, pGE-SABP2-2 and pGE-SABP2-3, respectively. *CsSABP2-1^V18A^* was synthesized by PCR using *CsSABP2-1* sequence and CsSABP2-1^V18A^-f/SABP2-1^V18A^-r as template and primers, respectively. The obtained *CsSABP2-1*, *CsSABP2-1^V18A^*, *CsSABP2-2* and *CsSABP2-3* sequence were confirmed by Sanger sequencing. To construct pPIC9K-SABP2 vectors to express CsSABP2 protein in yeast, four *CsSABP2* genes were amplified from the pGE-SABP2-1, pGE-SABP2-1^V18A^, pGE-SABP2-2 and pGE-SABP2-3 vectors with the primers pPIC9K-CsSABP2-1-f/pPIC9K-CsSABP2-1-r, pPIC9K-CsSABP2-1^V18A^-f/pPIC9K-CsSABP2-1^V18A^-r, pPIC9K-CsSABP2-2-f/pPIC9K-CsSABP2-2-r and pPIC9K-CsSABP2-3-f/pPIC9K-CsSABP2-3-r (**Supplementary Table S2**), respectively, and subsequently cloned into the yeast expression vector pPIC9K. The plant expression p35S::CsSABP2 vectors were constructed by unloading *CsSABP2* fragment with *Sma*I/*Sal*I from pGE-SABP2 vectors and inserted into the vector pLGN (Zou et al., 2017). In the vectors, *CsSABP2* expression was regulated by the strong 35S promoter.

### Bioinformatics analysis

Using Geneious 4.8.5 software to find the open reading frame (ORF) of *CsSABP2* gene. Protein conserved domain analysis was performed with the online software Pfam35.0 (http://pfam.xfam.org/). BLASTx was performed to find homologous amino acid sequences of CsSABP2 on NCBI website (http://blast.ncbi.nlm.nih.gov/Blast.cgi). MEGA7.0 (Kumar et al., 2016) was used to construct the phylogenetic tree of *CsSABP2* based on the neighbor-joining method.

### Protein expression, purification and enzyme activity analysis

pPIC9K-SABP2 plasmids were introduced into *Pichia pastoris* GS115 to produce CsSABP2 proteins. The purity of CsSABP2 proteins was assessed using SDS-PAGE electrophoresis. Protein concentrations were measured using the Bradford protein assay method (Bradford, 1976). The enzyme activity of CsSABP2 proteins was determined using the protocol described by Kumar and Klessig (2003). The assays were performed as follows: a 50 μl reaction mixture contained 50 mM MeSA substrate and 1 µg of CsSABP2 protein, and in the SA affinity reaction, an additional 50 mM SA substrate also was added to reaction. The reaction was incubated at 25 °C for 30 min, and then was halted by 200 µl ethyl acetate. SA production in the reaction were determined using plant SA ELISA (enzyme-linked immunosorbent assay) kits (Jiweibio, Shanghai, China). The MeSA esterase and SA affinity activities of CsSABP2 were expressed as nmol synthesized SA per minute per microgram CsSABP2 (nM. min^-1^µg^-1^). The experiment was performed in triplicate.

### Citrus transformation

The p35S::SABP2 vectors were transformed into *Agrobacterium* strain EHA105 for citrus transformation. Epicotyls from Wanjincheng orange seedlings served as explants for the transformation experiments (Peng et al., 2021). Transgenic shoots were confirmed through GUS staining and PCR analysis. Total genomic DNA was extracted from leaves with a plant genomic DNA extraction kit (Aidlab, Beijing, China). Specific primer pairs (**Supplementary Table S2**) were used to amplify specific gene products. The predicted products of *SABP2-1*, *SABP2-1^V18A^*, *SABP2-2* and *SABP2-3* were 804-, 804-, 792- and 819 bp long, respectively. PCR reactions were conducted using the following protocol: in 30 cycles of 94 °C/1 min, 58 °C/45s, 72 °C/1 min. Confirmed transgenic shoots were micrografted onto *in vitro* seedlings of Troyer citrange (*Poncirus trifoliata* (L.) Raf. × *Citrus sinensis* Osbeck) to facilitate shoot recovery. WT and transgenic plants were then grafted onto three-years old Troyer citrange seedlings and cultivated in a greenhouse at 28°C, 60% RH, and a 16 h photoperiod providing illumination of 45 µmol m^-2^ s^-1^.

### RNA extraction and RT-qPCR analysis

Total RNA of WT and transgenic plants was extracted from leaf tissues using an EASYspin Plant RNA Extraction Kit (Aidlab, Beijing, China). The extracted RNA was subsequently reversely transcribed into cDNA using a reverse transcription kit (TaKaRa, Tokyo, Japan). Gene expression was detected using the SYBR Prime qPCR Kit (Bioground Biotech, Chongqing, China) in a CFX96TM Real-Time System (Novogene, China). The PCR reactions were performed as follows: an initial pretreatment (95°C for 5 min) followed by 40 amplification cycles (94°C for 20 s; 60°C for 60 s). All primers used in the qRT-PCR experiments are detailed in **Supplementary Table S2**. *GAPDH* (Mafra et al., 2012) gene was served as an internal reference to normalize target gene expression. Relative expression levels were determined using the relative quantification method (2−ΔΔCt) (Livak and Schmittgen, 2001) with wild-type plants (WT) as control. All experiments were performed in triplicate.

### Measurement of SA, MeSA and H_2_O_2_ contents in citrus

Fresh tissues from fully mature leaves were used to extract SA, MeSA and H_2_O_2_. A total of 5 mL of extraction solution was added to 0.5 g (accurate to 0.0001 g) of the sample. The homogenate was shaken for 2 minutes in a mixture of isopropyl alcohol, water, and formic acid (80:19:1), and then subjected to ultrasonic extraction for 30 min at 4 °C. The supernatant was separated from the mixture by centrifuging at 10,000 g for 10 min at 4 °C. The nitrogen was reduced in the aqueous phase to 2 mL and diluted twofold with methanol. The extraction was then filtered through a 0.22-μm membrane. The levels of SA and MeSA contents in the isolations were measured using UPLC MS/MS at Chongqing Tengxin Biotechnology Co., Ltd (Chongqing, China). Also, SA and MeSA contents in citrus were measured as described by Zou et al. (2021): 0.5 g of fresh tissues from fully mature leaves were used to extract SA and MeSA, and then SA and MeSA levels were determined using the SA and MeSA ELISA (plant enzyme-linked immunosorbent assay) kits (Jiweibio, Shanghai, China) according to the manufacturer’s instructions, respectively.

According to the manufacturer’s instructions, hydrogen peroxide levels were measured using a Hydrogen Peroxide assay Kit (Solarbio, Beijing, China). A sample of 0.1 g (accurate to 0.0001 g) was added to 1 mL of Reagent 1 for homogenization in an ice bath. The supernatant was isolated by centrifuging at 8,000 g for 10 min at 4 °C. Then, 100ul of Reagent 2 and 200ul of Reagent 3 were added to the supernatant, and the mixture was centrifuged at room temperature for another 10 minutes. The precipitate was resuspended in Reagent 4, and the extracted H_2_O_2_ was measured at 415 nm.

SA, MeSA and H_2_O_2_ levels were determined based on the weight of the fresh leaves (µg/g FW). All tests were performed in triplicate.

### Evaluation of resistance to HLB in transgenic plants

The resistance of transgenic plants to HLB was assessed using the method outlined by Wang et al. (2023). Transgenic lines including WT plants were grafted with *Ca*Las-infected branches from Wanjincheng oranges. All the inoculated plants were kept in a controlled climate chamber at 28°C, with 60% relative humidity and a 16 h photoperiod. Disease development was regularly checked. Every two months after grafting, DNA was extracted from the midribs from three leaves (Zucol et al., 2006): The qPCR reaction was conducted in a final volume of 20 µL, which included 10 µL of the TaqProbe2×qPCR buffer, 7 µL H_2_O, 0.4 µL (10 mM·L^−1^) of HLBasf/r primers, 0.2 µL HLBp probe, and 2 µL DNA (10 ng·µL−1). The amplification protocol included an initial pretreatment step at 50°C for 2 min, followed by 40 amplification cycles consisting of 94°C for 3 s and 60°C for 30 s. The primer pairs for HLBas and HLBp are listed in **Supplementary Table S2**. The *Ca*Las population (*Ca*Las cells µg^−1^ of citrus DNA) was calculated based using the formula: y = -0.3101x + 12.09 (R^2^ = 0.99941), where x represents the quantity of DNA. The experiment was repeated three times.

### Evaluation of resistance to citrus canker in transgenic plants

Three-month old leaves of healthy WT and transgenic plants were challenged with *Xcc* using the method previously described by Peng et al. (2017). Briefly, small punctures were made in the leaves with a 0.5-mm pin, and each pinprick was inoculated with 1 µl *Xcc* suspension (1×10^5^ CFU ml^-1^). The petioles of inoculated leaves were wrapped in moist absorbent cotton. The treated leaves were placed in flat trays and were cultured at 28°C, 70% RH, and a 16h photoperiod with an illumination of 45 µmol m^−2^ s^−1^. Citrus canker development was recorded by photographing at 9 days post-inoculation (dpi), and diseased areas in leaf were estimated using ImageJ software (National Institutes of Health, Bethesda, MD). The disease index for the leaves of transgenic plants were calculated based on previously described methods (Peng et al., 2017). The experiment was conducted in triplicate.

### Statistical analyses

Statistical analyses of all data were conducted using SPSS V20 software (SPSS Inc., Chicago, IL, USA) and Prism V8 (GraphPad, USA). All experimental data are expressed as the means ± standard deviation (SD). Significant difference was established using Duncan’s test at a 0.05 level.

## Results

### Characterization of citrus *CsSABP2*

**Figure 1:**
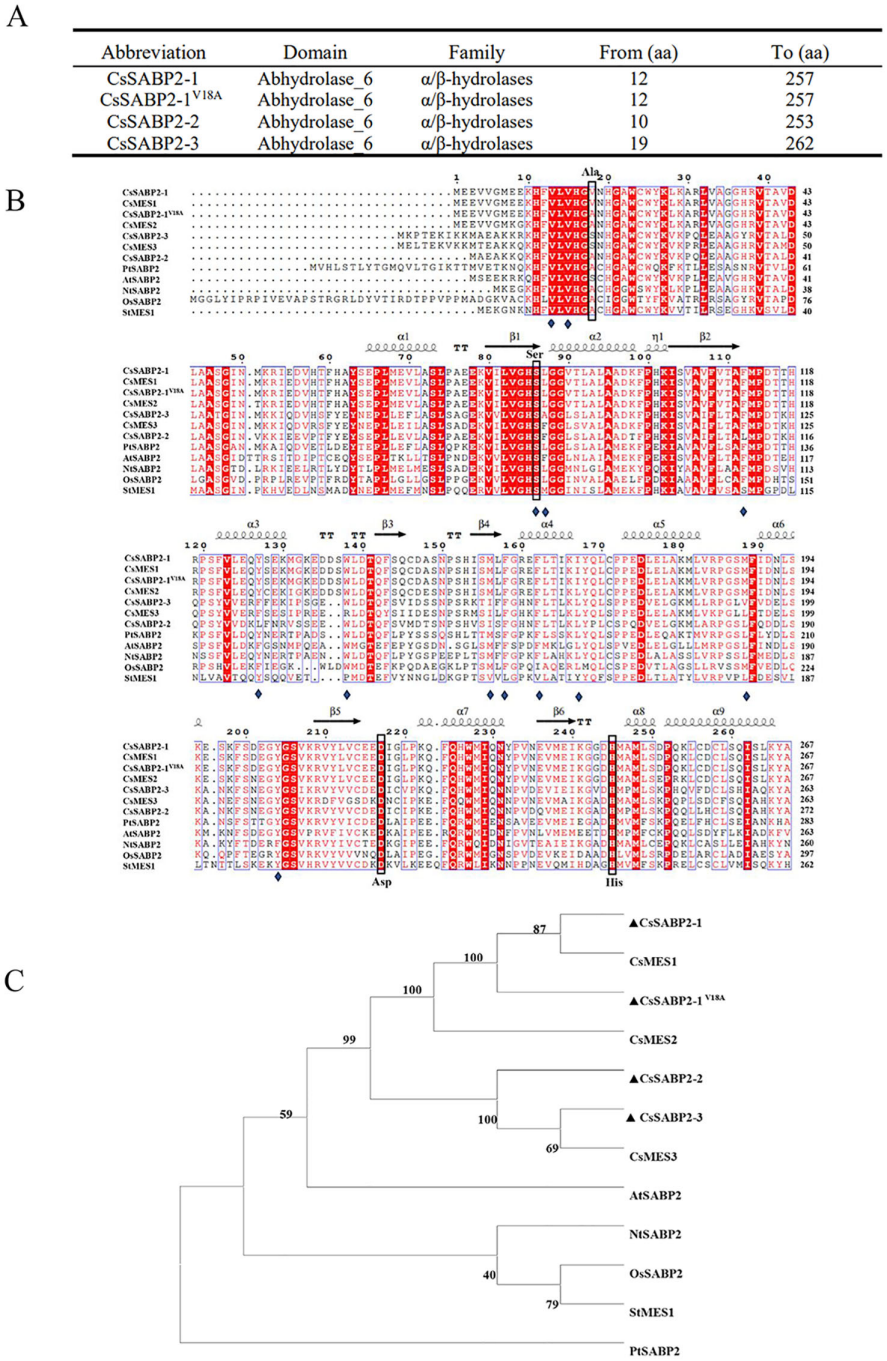
Analysis of CsSABP2 protein sequence. **(A)** Analysis of the conserved domains of the four SABP2 proteins in citrus. **(B)** Multiple sequence alignment of CsSABP2 with selected homologs. **(C)** A phylogenetic tree was constructed containing CsSABP2 and other selected homologs. Constructed by Neighbor Joining method with the full-length sequence of the proteins. The reliability of the tree was evaluated through 1000 bootstrap replicates, with the bootstrap values (%) shown above the branches. PtSABP2 (*Populus trichocarpa*, XP_002310754), AtSABP2 (*Arabidopsis thaliana*, NP_179943), StMES1 (*Solanum tuberosum*, NP_001275411), OsSABP2 (*Oryza sativa*, XP_015632670), NtSABP2 (*Nicotiana tabacum*, NP_001312442), CsMES1 (*Citrus sinensis*, KDO79352), CsMES2 (*Citrus sinensis*, XP_006466663), CsMES3 (*Citrus sinensis*, KDO48824). In **(B)**, α-helices is displayed as squiggles. β-strands are depicted as arrows, strict β-turns are indicated by TT, and strict α-turns are TTT. The folding type of protein secondary structures files is expressed as η. Blue diamonds represent SA-binding residues, while green and blue arrows highlight the secondary structural elements.

To investigate the role of citrus SABP2 genes against HLB and citrus canker, the coding sequences (CDS) of *CsSABP2-1*, *CsSABP2-2* and *CsSABP2-3* were cloned from Wanjincheng orange. To evaluate effect of the Valine-18 (Val-18) on CsSABP2-1 function, this amino acid was mutated to Alanine (Ala-18) to generate CsSABP2-1^V18A^. The sequencing results showed that the coding sequences (CDS) of *CsSABP2-1*, *CsSABP2-1^V18A^*, *CsSABP2-2* and *CsSABP2-3* were 804-, 804-, 792- and 819 bp, respectively (**Supplementary Table S1**). Bioinformatic analysis showed that all four proteins contained the conserved domain of Abhydrolase_6 hydrolase (**Figure 1A**). Abhydrolas_6 is a member of the α/β-hydrolase family with MeSA esterase activity (Li et al., 2019). Compared to tobacco SABP2 (NtSABP2), all CsSABP2-1, sSABP2-1^V18A^, CsSABP2-2 and CsSABP2-3 proteins contain the catalytic triad of Ser, His and Asp. The Asp residue in the catalytic triad is present in the secondary structure connecting α-helix (α7) and β-strand (β5) (**Figure 1B**). These data suggested that CsSABP2-1, CsSABP2-1^V18A^, CsSABP2-2 and CsSABP2-3 have SABP2 protease activity (Forouhar et al., 2005). Phylogenetic analysis showed that four CsSABP2 proteins are closely related to each other. Meanwhile, compared with the other five species, citrus SABP2 showed a closer relationship with a SABP2 from *Arabidopsis thaliana* (**Figure 1C**).

### Enzymatic activity of CsSABP2

**Figure 2:**
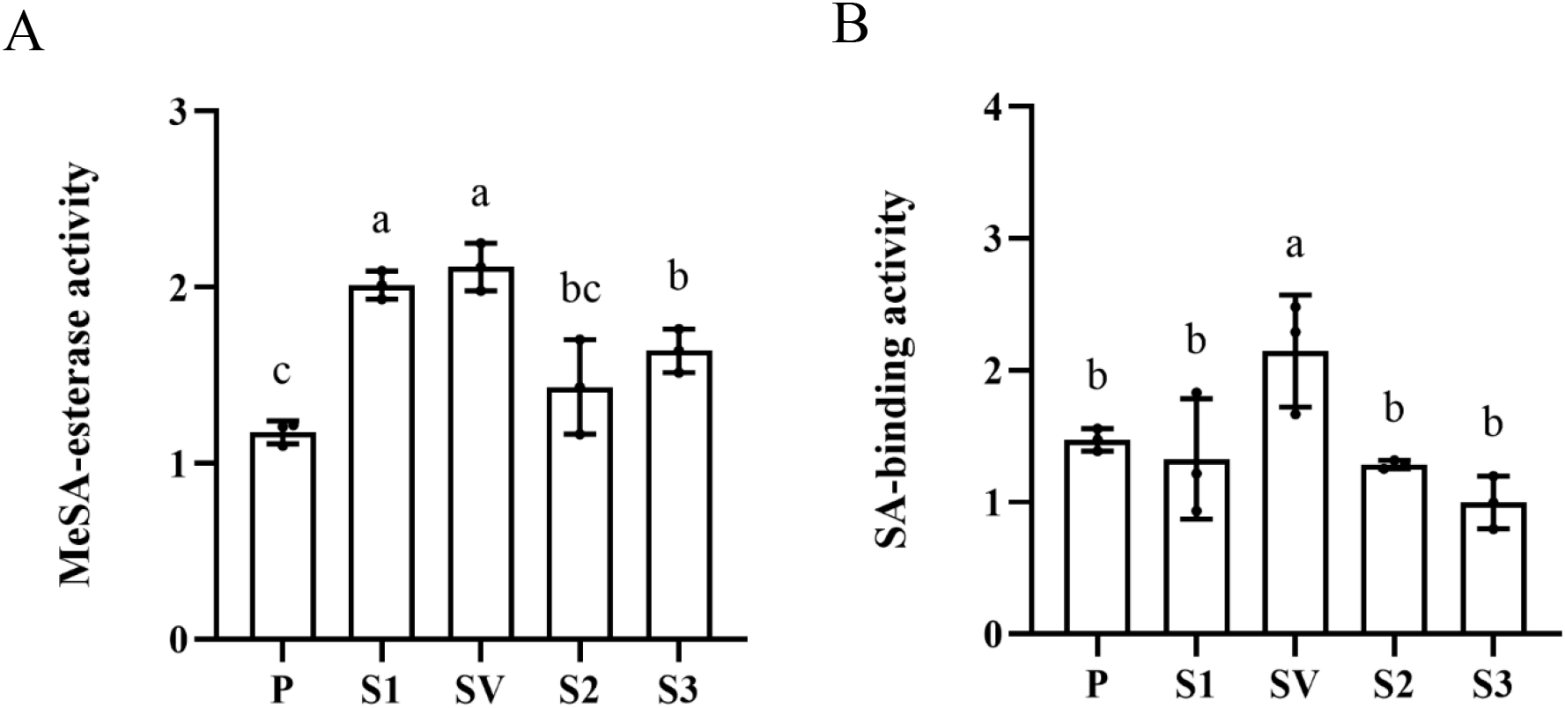
Enzyme activity of recombinant CsSABP2. Purification of recombinant CsSABP2 from Pichia pastoris eukaryotic expression system, and relevant enzymatic reactions were performed *in vitro*, and SA content of the mixture after reaction was determined to measure enzyme activity of recombinant CsSABP2. **(A)** MeSA-esterase activity of recombinant CsSABP2. **(B)** Inhibitory effect of SA on MeSA-esterase activity of recombinant CsSABP2. The MeSA-esterase activity expressed as nmol synthesized SA per minute per microgram CsSABP2 (nM. min^-1^µg^-1^). Different letters above the bars indicate significant differences from the pPIC9K control, as determined by Duncan’s test (p < 0.05, n = 3). P, pPIC9K; S1, pPIC9K-CsSABP2-1; SV, pPIC9K-CsSABP2-1^V18A^; S2, pPIC9K-CsSABP2-2; S3, pPIC9K-CsSABP2-3.

Four *CsSABP2* genes were separately transformed into yeast Pichia pastoris strain GS115 to generate the CsSABP2 recombinant protein (**Supplementary Figure S2**). Using MeSA as substrate, enzymatic activity analysis showed that SA productions in all four CsSABP2 recombinant protein treatments were significantly higher than that in empty pPIC9K control (**Figure 2A**), indicating that four CsSABP2 proteins can catalyzes the conversion of MeSA to SA. Among them, CsSABP2-1 and CsSABP2-1^V18A^ proteins had stronger esterase activity. It can be concluded that the mutation of Val-18 to Ala-18 of CsSABP2-1 does not affect its esterase activity, although there was a slight difference in esterase activity between CsSABP2-1 and CsSABP2-1^V18A^ proteins (**Figure 2A**).

It has been shown that SA binds the active site pocket of SABP2 to inhibit its MeSA esterase activity (Park et al., 2007). Here, effects of SA on MeSA esterase activity of four CsSABP2 proteins were also determined by adding SA into the above catalytic reaction (**Figure 2B**). When SA was added into the reaction, SA content in the CsSABP2-1^V18A^ treatment significantly increased compared with empty pPIC9K control, while no significant change in SA contents were detected in the CsSABP2-1, CsSABP2-2, CsSABP2-3 and empty pPIC9K control treatments. The results showed that SA completely inhibited the MeSA esterase activity of CsSABP2-1, CsSABP2-2 and CsSABP2-3 except for CsSABP2-1^V18A^, indicating that the mutation of Val-18 to Ala-18 eliminated the inhibition of SA on CsSABP2-1.

### Generation of transgenic citrus plants overexpressing *CsSABP2*

**Figure 3:**
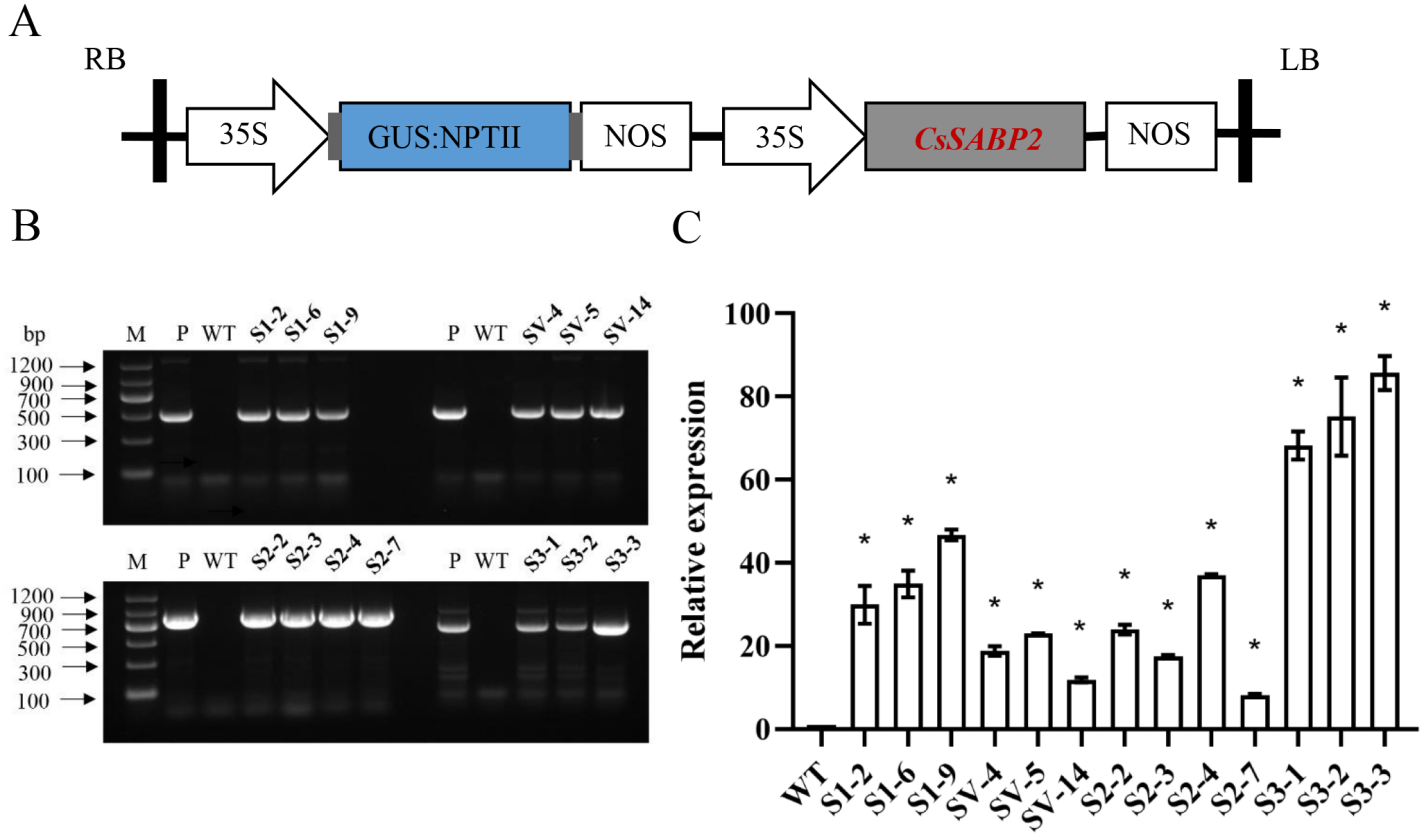
Identification of transgenic Wanjincheng oranges overexpressing *CsSABP2*. **(A)** The structure of the plasmid utilized for citrus transformation is depicted. The 35S promoter refers to the tobacco cauliflower mosaic virus 35S promoter; GUS: NPTII, represents the fusion of the β-glucuronidase and neomycin phosphotransferase genes, which are used for the screening of citrus transformants; nos, nos terminator; LB and RB refer to the left and right borders, respectively. **(B)** PCR confirmation of transgenic plants. **(C)** Relative expression of *CsSABP2* transgene in transgenic plants by qRT-PCR, normalized to *CsGAPDH* (Mafra et al., 2012). * above the bars represent significant differences from WT based on Duncan’s test (P < 0.05, n=3). WT, wild-type plants; S1-#, *CsSABP2-1* transgenic plants; SV-#, *CsSABP2-1^V18A^* transgenic plants; S2-#, *CsSABP2-2* transgenic plants; S3-#, *CsSABP2-3* transgenic plants.

To determine roles of CsSABP2 in resistance to HLB and citrus canker, four p35S::CsSABP2 expression cassettes (**Figure 3A**) were separately introduced into Wanjincheng oranges by *Agrobacterium*-mediated epicotyl transformation. Totally, 13 transgenic plants were identified by GUS staining and PCR (35S::CsSABP2-1: S1#, 35S::CsSABP2-1^V18A^: SV#, 35S::CsSABP2-2: S2# and 35S::CsSABP2-3: S3#) (**Figure 3B**). qRT-PCR further confirmed that the higher expression levels in all the S1#, SV#, S2# and S3# transgenic plants, when compared to WT plants (**Figure 3C**).

### Overexpression of *CsSABP2* in transgenic plants alters the accumulation of SA and MeSA

**Figure 4:**
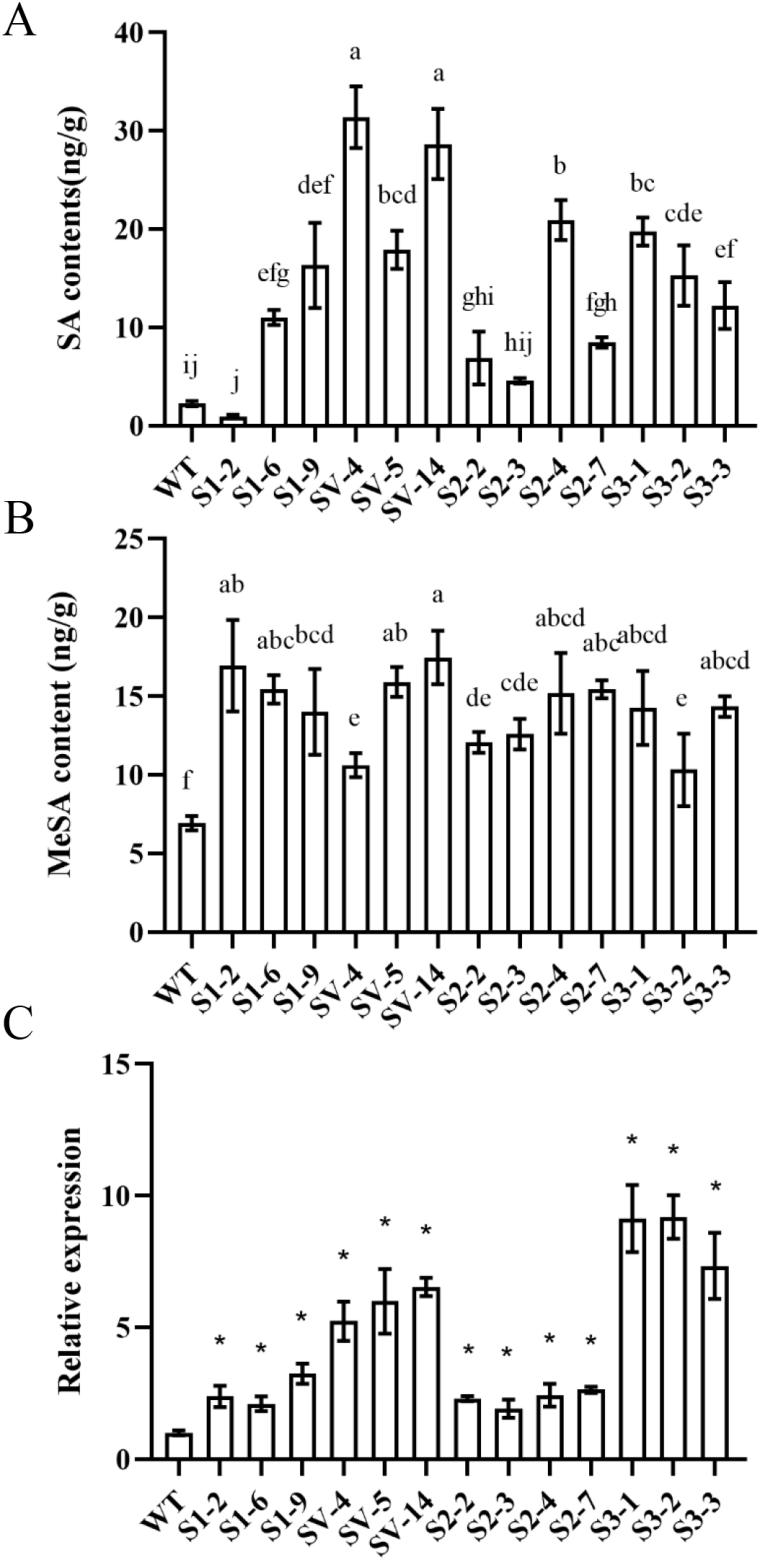
Characteristics of SA **(A)** and MeSA **(B)** contents and RT-qPCR analysis of *SAMT1* expression **(C)** in transgenic plants. SA and MeSA contents in the fully mature leaves from transgenic and WT plants were determined by UPLC MS/MS analysis. Different letters or * above the bars indicate significant differences from the wild-type (WT) as determined by Duncan’s test (P < 0.05, n = 3). SA, salicylic acid; MeSA, methyl salicylate. WT, wild-type plants; S1-#, CsSABP2-1 transgenic plants; SV-#, CsSABP2-1^V18A^ transgenic plants; S2-#, CsSABP2-2 transgenic plants; S3-#, CsSABP2-3 transgenic plants.

To understand the effects of *CsSABP2* overexpression on SA and MeSA accumulation, we measured the levels of SA and MeSA in healthy transgenic plants by UPLC MS/MS (**Figures 4A, B**). Compared with the WT control, most of transgenic plants had significantly increased SA contents except for the S1-2 transgenic lines overexpressing *CsSABP2-1* and the S2-2 and S2-3 transgenic line overexpressing *CsSABP2-2*. Further, there were the highest SA contents in *CsSABP2-1^V18A^* transgenic plants. All the *CsSABP2* transgenic plants had significantly increased MeSA, compared to WT control. These data implied that all CsSABP2 have esterase activity in citrus. We also estimated the expression of the *CsSAMT1* gene in transgenic plants using qRT-PCR (**Figure 4C**). Compared with the WT control, *CsSAMT1* was significantly upregulated in all the *CsSABP2* transgenic plants, revealing that MeSA increase in transgenic plants is due to the increased expression level of *CsSAMT1*. Based on the data, S1-6, S1-9, SV-4, SV-5, SV-14, S2-4, S2-7, S3-1, S3-2 and S3-3 lines containing high SA and MeSA were selected to be investigated in the following tests.

### Effects of *CsSABP2* overexpression on H_2_O_2_ accumulation in transgenic plants

**Figure 5:**
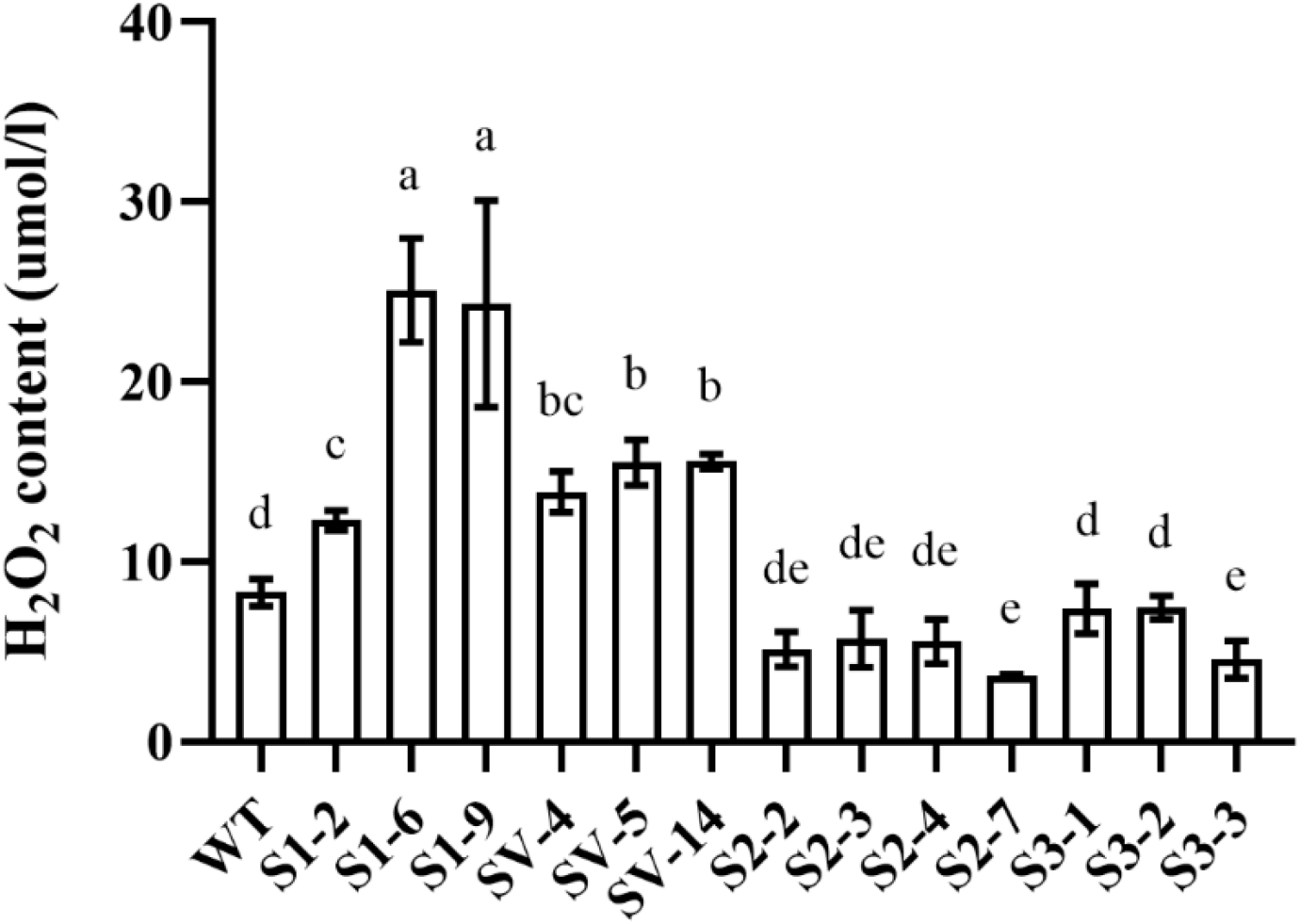
H_2_O_2_ contents in transgenic plants. WT, wild-type plants; S1-#, CsSABP2-1 transgenic plants; SV-#, CsSABP2-1^V18A^ transgenic plants; S2-#, CsSABP2-2 transgenic plants; S3-#, CsSABP2-3 transgenic plants. Different letters above the bars indicate significant differences from the wild-type (WT) as determined by Duncan’s test (P < 0.05, n = 3).

Next, we analyzed H_2_O_2_ contents and showed that significantly increased H_2_O_2_ in S1-# and SV-# lines, among which the H_2_O_2_ contents in the S1-6 and S1-9 line increased by more than 3 times, when compared to WT. H_2_O_2_ contents were reduced in all the S2-# and S3-# transgenic plants, but with little significance (**Figure 5**).

### Overexpressing CsSABP2 enhances tolerance to HLB in transgenic plants

**Figure 6:**
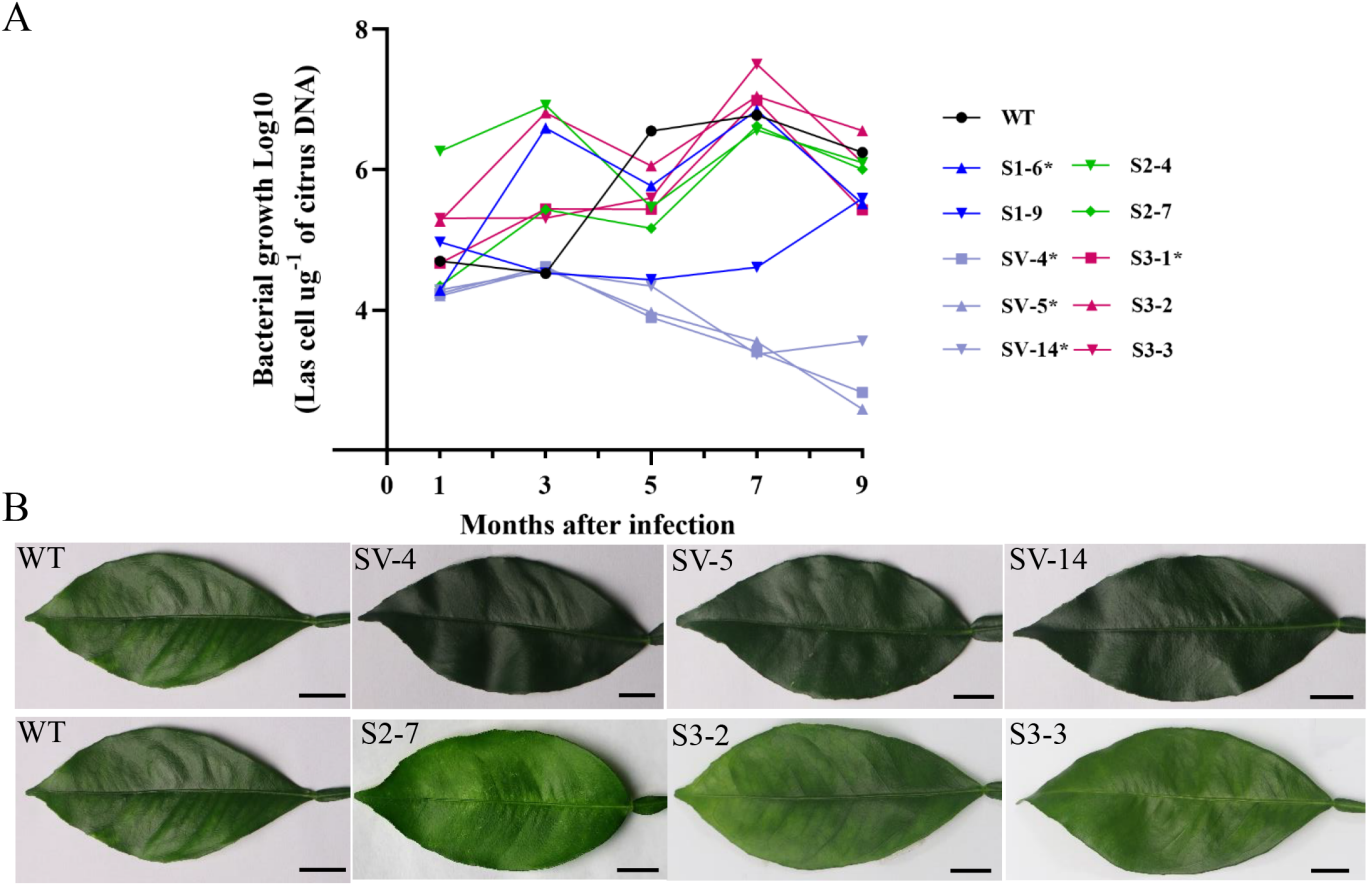
Evaluation of Citrus Huanglongbing (HLB) tolerance in transgenic citrus plants grown in a greenhouse. **(A)** Quantitative analysis of *CaLas* growth at 1, 3, 5, 7 and 9 months after infection (MAI). The bacterial populations [log_10_ (*CaLas* cells µg -1 of citrus DNA)] were determined using qPCR. Each column means standard deviation of three independent tests. * represents significant differences from the WT based on Duncan’s test (P < 0.05, n = 3) at 9 MAI. **(B)** HLB symptoms in the transgenic plants and WT plants at 7 MAI. WT, wild type; OE-#, transgenic plants. S1-#, *CsSABP2-1* transgenic plants; SV-#, *CsSABP2-1^V18A^* transgenic plants; S2-#, *CsSABP2-2* transgenic plants; S3-#, *CsSABP2-3* transgenic plants.

To evaluate resistance to HLB in the overexpressing *CsSABP2* transgenic plants, two-year-old transgenic plants including the WT control were infected by grafting branches containing the *Ca*Las pathogen. Detection of *Ca*Las in leaf tissues of plants by qPCR at one, three, five, seven and nine months after infection (MAI). Compared with WT plants, at 1 to 3 MAI, the S2-# line had significantly higher titers of *Ca*Las while the others showed no significant changes in *Ca*Las growth. After 5 MAI, *Ca*Las growth had no significant changes in S1-#, S2-# and S3-# lines compared to WT lines, but in all the SV-# lines it was significantly slower than in WT lines (**Figure 6A**). At 9 MAI, the contents of *Ca*Las of the SV-# lines reduced by 1.7-2.2 times, compared to WT control. Mottled yellow symptoms were initially observed in the leaves of WT plants at 7 MAI. We noticed that the transgenic lines S2-# and S3-# showed similar symptoms, specially, the S2-7, S3-2 and S3-3 lines were identified as the most severe HLB symptoms, which showed prominent veins. No obvious symptoms were detected in most of the S1-# lines and all the SV-# lines (**Figure 6B** and **Suplementary Figure S2**). Our data demonstrated that the overexpression of *CsSABP2-1^V18A^* enhance the tolerance to HLB caused by *Ca*Las in Wanjincheng oranges.

### Overexpressing *CsSABP2* enhances resistance to citrus canker in transgenic plants

**Figure 7:**
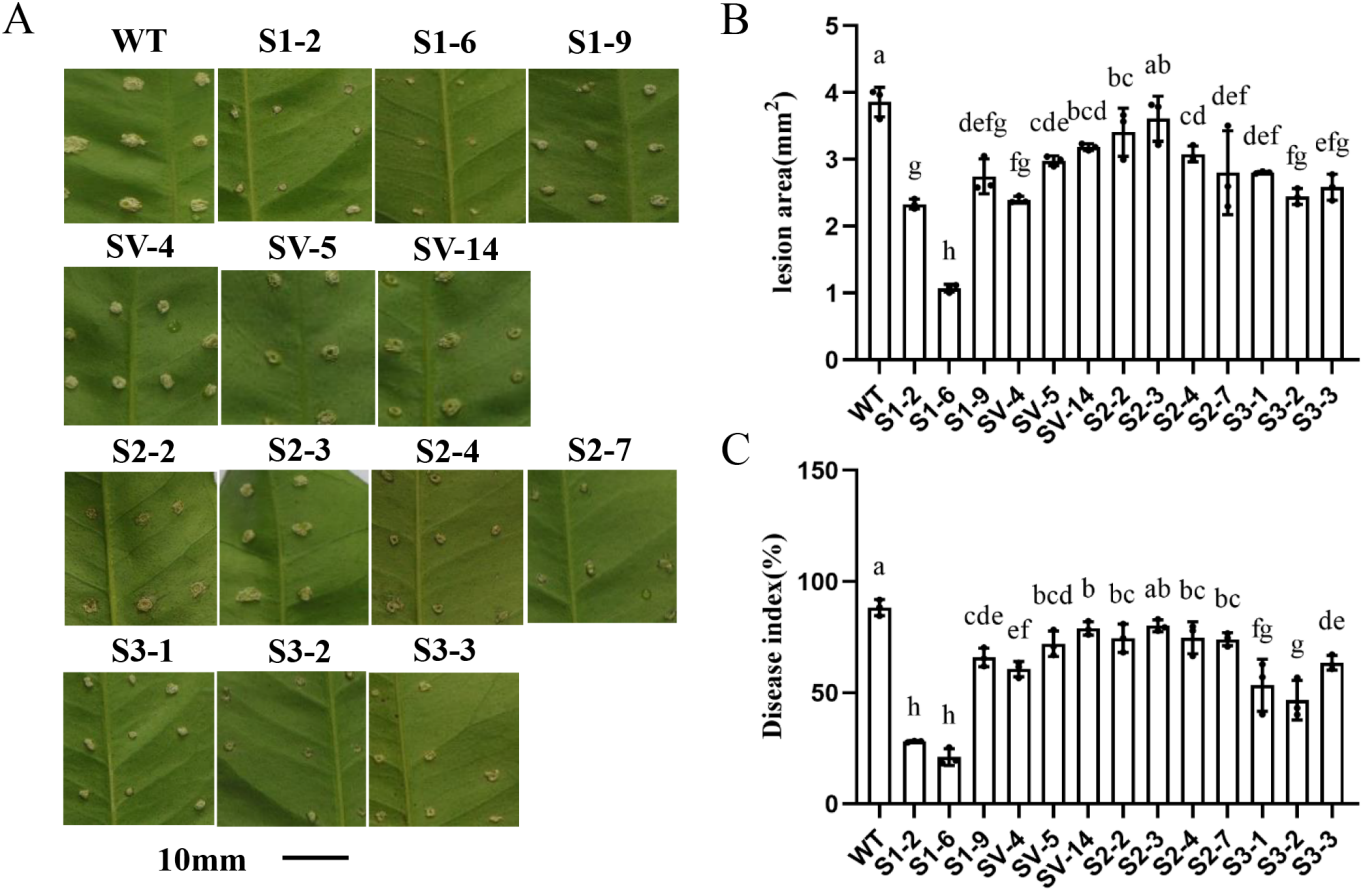
Evaluation of resistance to citrus canker in *CsSABP2*-overexpressing ‘Wanjincheng’ oranges. Fully expanded (about three-months old) leaves of transgenic and WT plants were inoculated with 1×10^8^ CFU ml^-1^ *Xanthomonas citri* subsp. *citri* (*Xcc*). Citrus canker symptoms **(A)**, Lesion areas **(B)** and disease indices **(C)** in leaves were counted 9 days post-inoculation (dpi). Each column represents the standard deviation from three independent tests. Different letters above the bars indicate significant differences from the wild-type (WT) as determined by Duncan’s test (P < 0.05, n = 3) at 9 dpi. WT, wild-type plants; S1-#, *CsSABP2-1* transgenic plants; SV-#, *CsSABP2-1^V18A^* transgenic plants; S2-#, *CsSABP2-2* transgenic plants; S3-#, *CsSABP2-3* transgenic plants.

To evaluate the resistance of transgenic plants to citrus canker, leaves from both transgenic and WT plants were inoculated with *Xcc* using the pinprick method (Peng et al., 2017). The diseased symptoms were determined 9 days post-inoculation (dpi). Lesions in most *CsSABP2*-overexpressing lines were significantly smaller than those in WT plants (**Figures 7A, B**). The disease indices on leaves of these transgenic plants were also reduced significantly compared with WT plants (**Figure 7C**). Among all of these *CsSABP2*-overexpressing lines, the *CsSABP2-1* and *CsSABP2-3* overexpressing lines exhibited very small lesions. Based on these results, *CsSABP2* overexpression enhances resistance to *Xcc* in ‘Wanjincheng’ oranges.

### Effects of *CsSABP2* overexpression on SAR responses in transgenic plant

**Figure 8:**
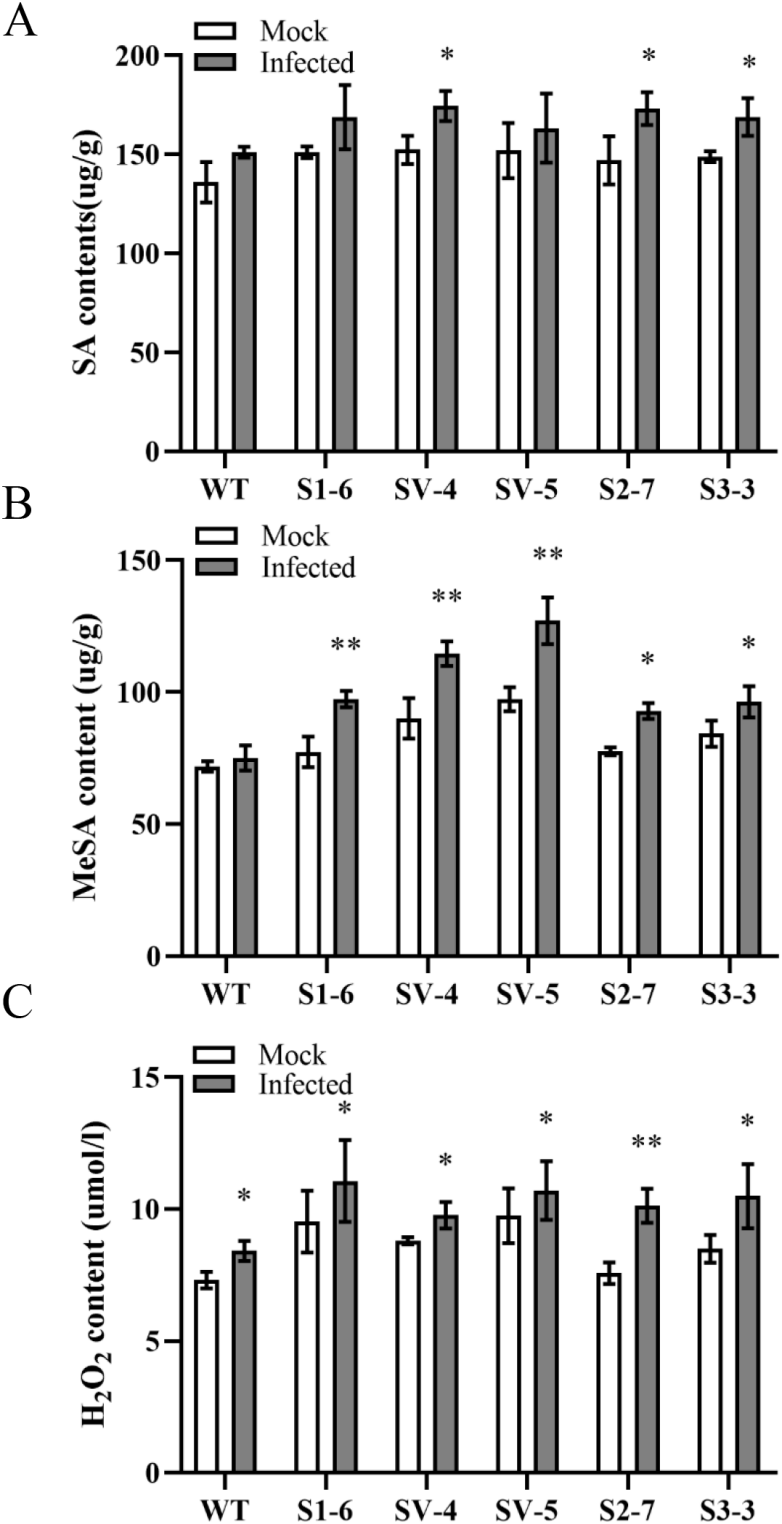
Characteristics of SA **(A)**, MeSA **(B)** and H_2_O_2_ **(C)** in response to *Xanthomonas citri* pv. *Citri* (*Xcc*) infection. The fully mature leaves from transgenic and WT plants were infiltrated by 1×10^5^ CFU ml^-1^ *XCC*. At two days after inoculation, SA and MeSA and H_2_O_2_ contents in the treated leaves were determined by the plant enzyme-linked immunosorbent assay (ELISA) and Hydrogen Peroxide assay Kits, respectively. SV-#, CsSABP2-1^V18A^ transgenic plants; S2-#, CsSABP2-2 transgenic plants; S3-#, CsSABP2-3 transgenic plants; SA, salicylic acid; MeSA, methyl salicylate; Mock, sterile water inoculation; Infected, *XCC* inoculation. The asterisks above the bars indicate significant differences from the control group (Mock), as determined by the T-test. A single asterisk indicates significance at the 0.05 level, while two asterisks indicate significance at the 0.01 level (n = 3).

To understand how *CsSABP2* affects SAR responses, we further investigated characteristics of SA, MeSA and H_2_O_2_ in response to *Xcc* inoculation by the infiltration method (Long et al., 2019). Two days after infection, SA showed increased levels in all the plants compared to mock controls as well as its contents in *Xcc*-infected transgenic plants were higher than that of WT control (**Figure 8A**). MeSA levels were significantly evaluated by *Xcc* inoculation in all the transgenic plants compared to their controls (**Figure 8B**). No change in MeSA was detected in WT plant. Among them, the SV-4 and SV-5 transgenic plants exhibited the fastest increase of MeSA in response to *Xcc*. The H_2_O_2_ levels in both transgenic and WT plants were also significantly evaluated by *Xcc*, compared to mock controls although the increased levels in transgenic plants were higher than those in the WT (**Figure 8C**). The results demonstrated that *CsSABP2* overexpression can enhance SA, MeSA and H_2_O_2_ accumulation in response to pathogen infection.

**Figure 9:**
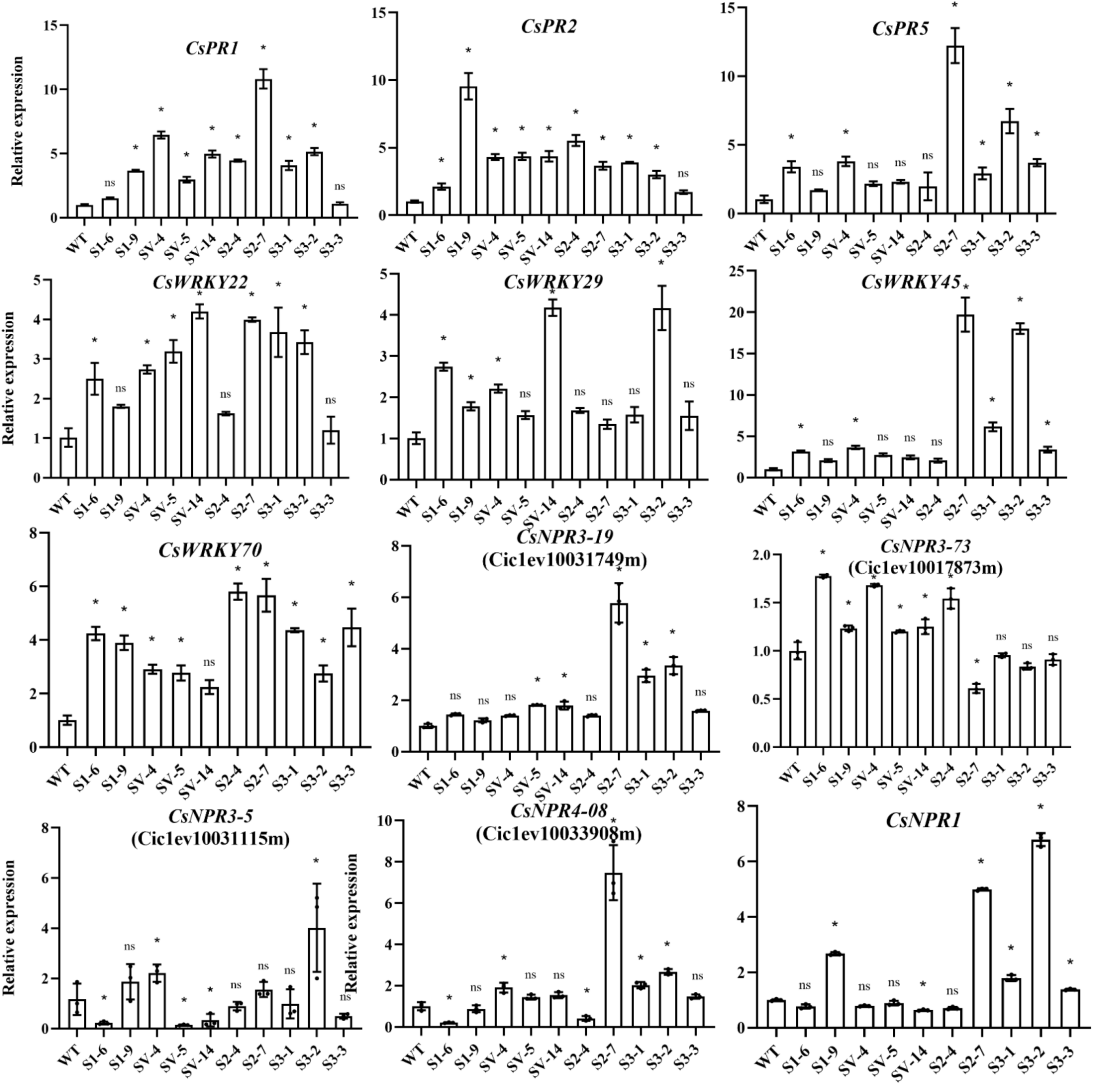
RT-qPCR analysis of the expression level of immunity-related genes in transgenic plants. The relative expression of *CsPR1*, *CsPR2*, *CsPR5*, *CsWRKY22*, *CsWRKY29*, *CsWRKY45*, *CsWRKY70*, *CsNPR1*, *CsNPR3-5*, *CsNPR3-19*, *CsNPR3-73* and *CsNPR4-08* in transgenic lines were measured using *GAPDH* (Mafra et al., 2012) gene as an internal reference, compared with the wild-type control. * and ns on top of the bars represent significant differences and no differences from WT, respectively, based on Duncan’s test (P < 0.05, n=3). WT, wild-type plants; S1-#, *CsSABP2-1* transgenic plants; SV-#, *CsSABP2-1^V18A^* transgenic plants; S2-#, *CsSABP2-2* transgenic plants; S3-#, *CsSABP2-3* transgenic plants.

We also estimated the expression of some immunity-related key genes in transgenic plants by qRT-PCR, including three SAR marker genes (*CsPR1*, *CsPR2*, *CsPR5*), four *WRKY* genes (*CsWRKY45*, *CsWRKY70*, *CsWRKY22* and *CsWRKY29*), five *NPR1*-like genes (*CsNPR1* for Cs_ont_4g011050, *CsNPR3-73* for Ciclev10017873m, *CsNPR3-15* for Ciclev10031115m, *CsNPR3-49* for Ciclev10031749m and *CsNPR4-08* for Ciclev10033908m) (Long et al., 2019; Pang et al., 2020; Wu et al., 2021; Du et al., 2022). The data displayed that these genes showed remarkedly different expression profiles among *CsSABP2-1*, *CsSABP2-2*, *CsSABP2-3* and *CsSABP2-1^V18A^* transgenic plants, when compared with the WT control (**Figure 9**). We noted that *CsPR1*, *CsPR2*, *CsWRKY22* and *CsNPR3-73* had significantly increased expression in all the *CsSABP2-1^V18A^* transgenic plants with the strongest HLB tolerance (**Figure 6**), suggesting that these genes play an important role in *CsSABP2-1^V18A^*-mediated resistance to HLB. *CsPR2*, *CsWRKY29*, *CsWRKY70* and *CsNPR3-73* had also significantly increased expression in the *CsSABP2-1* transgenic plants. Additionally, *CsPR5*, *CsWRKY45*, *CsWRKY70*, and *CsNPR1* were significantly upregulated in all the *CsSABP2-3* transgenic plants. The two kinds of transgenic plants showed higher resistance to *Xcc*, suggesting that these genes involved in *CsSABP2-1* or/and *CsSABP2-3*-mediated resistance to citrus canker. Additionally, *CsPR1*, *CsPR2* and *CsWRKY70* were significantly upregulated in both the *CsSABP2-2* transgenic plants. The expression of *CsPR1*, *CsPR2*, and/or *CsPR5* was significantly upregulated by *CsSABP2-1*, *CsSABP2-2*, *CsSABP2-3* and/or *CsSABP2-1^V18A^* overexpression, indicated that overexpression of the four *CsSABP2* genes can activate citrus SAR. Further, based on the data, we suggested that *CsPR2* and *CsNPR3-73* should be potential candidates for engineering SAR to HLB and citrus canker.

## Discussion

### SAR has great potential to engineer broad-spectrum resistance in citrus

At present, the global citrus industry is facing the compounded challenges of HLB and citrus canker (Gabriel et al., 2020). Still now, a great number of genes from citrus or other species have been applied to improve citrus canker through transgenic and gene edit technology (Peng et al., 2017; Deng and Ma, 2020; Conti et al., 2021). Until recently, a few genes have been identified to be potential to enhance citrus tolerance to HLB disease (Peng et al., 2021; Nascimento et al., 2022; Cheng et al., 2023). But these exiting studies have paid little to development of broad-spectrum resistance to HLB and citrus canker. Breeding broad-spectrum resistance is one of the most efficient strategies for controlling the two diseases. For example, we recently demonstrated that expression of the LasLYS2 endolysin gene from *Ca*Las confers dual resistance to HLB and citrus canker in Carrizo citrange (Citrus sinensis × Poncirus trifoliata) (Xu et al., 2023). Overexpressing a plant thionin enhances resistance to citrus canker and HLB in Carrizo citrange (Hao et al., 2016).

Manipulating plant SAR can confer broad-spectrum resistance against pathogens. We previously found that *CsSAMT1* overexpression in Wanjincheng oranges increased tolerance to HLB (Zou et al., 2021), meanwhile Nascimento et al. (2022) displayed that this gene provides resistance to citrus canker in Hamlin and Valencia sweet oranges (*C. sinensis*), indicating that engineering the conversion of SAR signal from SA to MeSA can simultaneously improve resistance to HLB and citrus canker. In the uninfected tissue, defense-inactive MeSA is required to be converted to active SA to trigger SAR-mediated resistance and SABP2 is the key enzyme for transferring back MeSA to SA (Park et al., 2007). The crucial function of SABP2 in SAR has been widely understood in many species (Vlot et al., 2021). However, the roles of citrus-derived SABP2 in citrus SAR against HLB and citrus canker is still unclear. Thus, in this study, we further investigated effects of engineering the conversion of SAR signal from MeSA to SA on resistance to HLB and citrus canker through overexpressing four *CsSABP2* genes in Wanjincheng oranges. Based on the data presented in the study, we concluded that this strategy is also effective to enhance citrus tolerance to HLB and citrus canker. Together, our and other researchers results demonstrated that SAR-mediated defense has great potential to improve broad-spectrum resistance in citrus breeding (Zhang et al., 2010; Dutt et al., 2015; Boscariol-Camargo et al., 2016; Zou et al., 2021; Nascimento et al., 2022).

### Effects of amino acid polymorphism of CsSABP2 on disease resistance of citrus

de Lima Silva et al. (2019) reported three *SABP2* homologue genes from Citrus sinensis: *CsMES1*, *CsMES2* and *CsMES3*. In our previous studies, three *SABP2* genes were cloned successfully from Wanjincheng orange (Zhao et al., 2021). *CsSABP2-1* is exactly *CsMES1*, *CsMES3* was highly homologous with *CsSABP2-3*. However, *CsMES2* is not found in Wanjincheng orange. The data indicated that SABP2 has abundant polymorphism in citrus. We also artificially synthesized *CsMES2* (named as *CsSABP2-1^V18A^* in this study) to investigate effect of Val-18 in the active site of *CsSABP2-1* on gene functions.

SABP2 belongs to α/β-hydrolase which has a highly conserved Ser-His-Asp catalytic triad motif (Holmquist, 2000; Forouhar et al., 2005). All CsSABP2-1, CsSABP2-1^V18A^, CsSABP2-2 and CsSABP2-3 have the catalytic triad motif. However, they showed different esterase activities *in vitro*. It was suggested that the polymorphism of the acid residue corresponding to Ala-13 in the active site of tobacco SABP2 influences the catalytic efficiency of CsSABP2 enzymes (de Lima Silva et al., 2019). In tobacco SABP2, Ala-13 forms a hydrogen bond with one of the oxygen atoms in the carboxyl group of SA during the catalytic reaction (Forouhar et al., 2005). The Ala-13 in tobacco SABP2 corresponds to Val-18 in CsSABP2-1, Ala-18 in CsSABP2-1^V18A^, Ala-16 in CsSABP2-2 and Ser-25 in CsSABP2-3, respectively (**Figure 1B**). the V18A and V18S mutation in CsMES1 causes lower catalytic efficiency (de Lima Silva et al., 2019). But, in our study, the V18A mutant has no significant effect on CsSABP2-1 activity, although CsSABP2-3 containing Ser-25 residue exhibited significant decreased MeSA esterase activity when compared to CsSABP2-1. In fact, CsSABP2-2 has Ala-16 residue, but its activity is still significantly lower than that of CsSABP2-1. Thus, it should be that other polymorphic residues in CsSABP2-1, CsSABP2-1^V18A^, CsSABP2-2 and CsSABP2-3 affected their esterase activities, which is required to be determined in future. Moreover, the mutation of Ala-13 to Leu-13 in NtSABP2 lacks SA-binding activity and SA-feedback inhibition (Park et al., 2007), indicating Ala-13 plays a key role in SA-binding activity of SABP2. It is also suggested that the Val-18 in CsMES decreases the SA/MeSA binding affinity (de Lima Silva et al., 2019). Our data revealed that the V18A mutation (CsSABP2-1^V18A^) of CsSABP2-1 eliminates inhibitory effect of SA on its esterase activity. This may be due to the V18A mutation increases the MeSA binding affinity, and as a result, represses SA binding, finally to favor CsSABP2-1^V18A^ esterase activity. However, CsSABP2-2 with Ala-16 and CsSABP2-3 with Ser-25 does not eliminate this role of SA. Hormone analysis revealed that the increased SA level in CsSABP2-1^V18A^ transgenic plants was obviously higher than that of CsSABP2-1, CsSABP2-2 and CsSABP2-3 transgenic plants, which was basically consistent with the result in the enzymatic activity analysis. This revealed that the Ala-18 residue plays crucial role in maintaining the MeSA esterase activity of CsSABP2 in citrus. Finally, resistance evaluation found that CsSABP2-1^V18A^ had dual resistance to HLB and citrus canker although CsSABP2-1 and CsSABP2-3 had higher resistance to citrus canker. But, CsSABP2-1, CsSABP2-2 and CsSABP2-3 had no visible resistance to HLB, further indicating Ala-18 residue is a key in CsSABP2-mediated resistance to HLB. Together, our data suggested that effects of the acid residue polymorphism on CsSABP2’s resistance are various in different proteins, which may be associated with protein structures.

### Maintaining homeostasis between SA and MeSA and moderately increasing H_2_O_2_ favor CsSABP2-mediated resistance in citrus

Several studies have shown that SA and MeSA-mediated innate defenses play vital roles in citrus response to HLB and canker (Aritua et al., 2013; Martinelli et al., 2013; Wang et al., 2016; Long et al., 2019). External application of SA and MeSA reduces HLB and citrus canker (Li et al., 2017; de Lima Silva et al., 2019; Cheng et al., 2023). Further, manipulating endogenous SA and MeSA levels confers tolerance to HLB and citrus canker (Zou et al., 2021; Nascimento et al., 2022). *CsSAMT1* overexpression increased both SA and MeSA levels, companied with enhanced tolerance to HLB in transgenic plants (Zou et al., 2021), indicating that maintaining homeostasis between SA and MeSA accumulation is beneficial for HLB tolerance of citrus (Long et al., 2019; Liu et al., 2023). In the presented study, *CsSABP2-1^V18A^* overexpression also promoted SA and MeSA accumulation simultaneously as well as enhanced resistance to HLB and citrus canker in transgenic plants. These data suggested that homeostasis between SA and MeSA accumulation is also important to engineer a broad-spectrum resistance in citrus. However, this study indicated that SA and MeSA had different roles in transgenic plants challenging to *Ca*Las and *Xcc*. It has been suggested that the basal level of SA is linked to the resistance of citrus varieties to HLB, and the increase in MeSA and the decrease in SA induced by *Ca*Las favors plant resistance (Liu et al., 2023). SA positively regulates callose deposition during pathogen infection (Yi et al., 2014). Over-deposition of callose in sieve elements is believed to be a pathogenic factor contributing to the greening symptoms (da Graça et al., 2016; Achor et al., 2020). Moreover, systemic and chronic callose deposition induced by *Ca*Las in sieve elements disrupts phloem transport function (Ma et al., 2022). MeSA has no inherent bioactivity, however, its accumulation can mitigate greening symptoms by reducing callose deposition during *Ca*Las infection (Long et al., 2019). Thus, we speculated that the increased MeSA in *CsSABP2-1^V18A^* transgenic plants can neutralize the adverse effects of SA meanwhile the higher SA conferred enhanced tolerance to HLB.

It is suggested that overaccumulation of ROS (such as H_2_O_2_) contributes to symptoms of HLB, including chlorosis and leaf discoloration (Pitino et al., 2017; Ma et al., 2022). *CsSABP2-1^V18A^* transgenic plants had increased H_2_O_2_ contents and synchronously showed strong tolerance to HLB, compared to *CsSABP2-2* and *CsSABP2-3* transgenic plants with similar levels of H_2_O_2_ to the WT control. *SABP2-1* transgenic plants had the highest levels of H_2_O_2_, that is also significantly higher than that of *SABP2-1^V18A^* transgenic plants. However, *SABP2-1* transgenic plants had no enhanced tolerance to HLB, indicating excessive levels of ROS is not beneficial for resistance to HLB. Further, *CsSABP2-2* and *CsSABP2-3* transgenic plants had not enhanced tolerance to HLB. Thus, the above data suggested that moderately increasing H_2_O_2_ is important for CsSABP2-mediated resistance to HLB in citrus.

We further investigated that how SA, MeSA and H_2_O_2_ responded to pathogen in transgenic plants. Still now, *Ca*Las can not be cultured *in vitro* and has a long-incubation period when it enters into plant (Xu et al., 2023). Moreover, the pathogen is not evenly distributed among infected different plants and tissues (Bové and Barros, 2006; Wang and Trivedi, 2013). In this study, to minimize the effect of these factors on the analysis of SA, MeSA and H_2_O_2_ contents, *Xcc* was used to investigate the response of SA, MeSA and H_2_O_2_ to pathogen through *in vitro* infiltration method. The data revealed that SA, MeSA and H_2_O_2_ rapidly accumulate in response to *Xcc* infection in transgenic plants (**Figure 8**). Especially, MeSA accumulation in the *CsSABP2-1^V18A^* transgenic plants was the highest among these transgenic plants. High MeSA accumulation induced by *Ca*Las facilitates citrus against HLB (Zou et al., 2021; Cheng et al., 2023; Liu et al., 2023). Together, the findings suggested that *CsSABP2* overexpression enhances SAR-mediated priming resistance and also further triggers SAR response to pathogen infection in citrus.

### CsSABP2 overexpression primes SAR-related transcription activities

Expression analysis of immunity-related genes demonstrated that CsSABP2 overexpression primed SAR-related transcription activities. NPR1 plays a central role in SA-dependent SAR defense through activating the transcription of downstream transcription factor genes (such as *WRKY70* and *WRKY45*), and subsequently which transcriptionally regulated defense-related genes (such as pathogenesis-related (PR) proteins *PR1*, *PR2* and *PR5* genes) (Li et al., 2004; Fu and Dong, 2013). The data showed that *CsSABP2-1*, *CsSABP2-2*, *CsSABP2-3* and *CsSABP2-1^V18A^* overexpression caused various expression profiles among the tested genes, this difference may be a potential reason causing the discrepancy of disease resistance among the four CsSABP2. SA triggers NPR1 activity through a dose-dependent manner (Boatwright and Pajerowska-Mukhtar, 2013), which finally affects the expression of downstream genes. Four CsSABP2s had different capacity of SA production and thus caused various gene expression levels in transgenic plants through SA dose-dependent regulation of NPR1 mediator. It is also clear that four *CsSABP2* overexpression induced one or two PR gene among *PR1*, *PR2* and *PR5*, indicating all the *CsSABP2*s can promote citrus SAR response.

Our gene expression analysis emphasized the important role of *CsPR2* and *CsNPR3-73* in CsSABP2-mediated broad-spectrum resistance to HLB and citrus canker. PR2 is predicted to encode a β-1,3-glucanase, an enzyme degrading the fungal cell wall (Datta and Muthukrishnan, 1999) or plant endogenous substrates to produce elicitors, thereby inducing defense responses (van Loon et al., 2006). More studies reveal that PR2 is an enzyme that catalyzes callose turnover and negatively affects callose deposition (Oide et al., 2013). In our recent study, Knockdown of CsCalS11, which is responsible for callose production, reduced callose deposition and symptoms in *Ca*Las-infected citrus (Yao et al., 2023), suggesting it is possible that engineering CsPR2 to inhibit callose deposition for improving citrus resistance to HLB. However, silencing CalS1 makes lemons more susceptible to *Xcc* (Enrique et al., 2011). Thus, increasing CsPR2 expression may be not resistant to citrus canker. NPR1 is the central regulator of SA-mediated defense while the NPR1-like homologies, *NPR3* and *NPR4*, serve as negative regulators to repress the expression of SA-responsive genes through promoting NPR1 degradation (Ding et al., 2018). Overexpressing *NPR1* exhibits enhanced resistance to HLB and citrus canker (Dutt et al., 2015; Boscariol-Camargo et al., 2016). Our previous study confirmed that a CsNPR1-like homology overexpression (named as *CsNPR3-19* here) improved the resistance of the Wanjincheng orange to HLB (Peng et al., 2021). However, *CsNPR1* and *CsNPR3-19* expression was not significantly affected by *CsSABP2* overexpression. But, the overexpression of all the four *CsSABP2* increased *CsNPR3-73* expression, suggesting *CsNPR3-73* has a potential role in regulating disease-resistance in citrus. Thus, how *CsPR2* and *CsNPR3-73* participated in CsSABP2-mediated SAR response is further understood in future studies.

## Conclusion

In summary, our study demonstrated that four *CsSABP2* genes had different roles in tolerance to HLB and citrus canker. *CsSABP2-1^V18A^* is a preferred candidate for conferring broad-spectrum resistance to HLB and citrus canker. The data also suggested the polymorphism of Val-18 in the active site of CsSABP2 plays a key role in CsSABP2 function, which provide meaningful references to engineering SABP2 for a broad-spectrum resistance of citrus. We highlighted that maintaining the homeostasis between SA and MeSA signals is important to confer a broad-spectrum resistance to HLB and citrus canker when manipulating citrus SAR. Further field trial is underway to investigate the resistance of these transgenic plants obtained in this study by exposure to free-flying ACPs.

## Data Availability

The datasets presented in this study can be found in online repositories. The names of the repository/repositories and accession number(s) can be found in the article/**Supplementary Material**.
