# Supplementary material for: Overexpressing CsSABP2 enhances tolerance to Huanglongbing and citrus canker in C. sinensis
Source: Front Plant Sci. 2024 Oct 8;15:1472155. doi: 10.3389/fpls.2024.1472155 (PMC11493644; doi:10.3389/fpls.2024.1472155)
Supplement: Supplementary file 1 [file DataSheet1.docx]

**Overexpressing *CsSABP2* enhances tolerance to Huanglongbing and citrus canker in *C. sinensis***

Liting Dong^a^, Shuang Chen^a^, Lanyue Shang^a^, Meixia Du^a^, Kaiqin Mo^a^, Shuwei Pang^a^, Lin Zheng^a^, Lanzhen Xu^a^, Tiangang Lei^a^, Yongrui He^a^, Xiuping Zou^a,^*

^a^ Integrative Science Center of Germplasm Creation in Western China (CHONGQING) Science City, Citrus Research Institute, Southwest University/National Citrus Engineering Research Center, Chongqing, People’s Republic of China

* Corresponding authors. E-mail addresses: [zouxiuping@cric.cn](mailto:zouxiuping@cric.cn)

**Table S1** The amino acid sequences of CsSABP2s from Wanjincheng orange

| Gene name | Sequences |
| --- | --- |
| CsSABP2-1 | MEEVVGMEEKHFALVHGVNHGAWCWYKLKARLVAGGHRVTAVDLAASGINMKRIEDVHTFHAYSEPLMEVLASLPAEEKVILVGHSLGGVTLALAADKFPHKISVAVFVTAFMPDTTHRPSFVLEQYSEKMGKEDDSWLDTQFSQCDASNPSHISMLFGREFLTIKIYQLCPPEDLELAKMLVRPGSMFIDNLSKESKFSDEGYGSVKRVYLVCEEDIGLPKQFQHWMIQNYPVNEVMEIKGGDHMAMLSDPQKLCDCLSQISLKYA* |
| CsSABP2-1^V18A^ | MEEVVGMEEKHFVLAHGANHGAWCWYKLKARLVAGGHRVTAVDLAASGINMKRIEDVHTFHAYSEPLMEVLASLPAEEKVILVGHSLGGVTLALAADKFPHKISVAVFVTAFMPDTTHRPSFVLEQYSEKMGKEDDSWLDTQFSQCDASNPSHISMLFGREFLTIKIYQLCPPEDLELAKMLVRPGSMFIDNLSKESKFSDEGYGSVKRVYLVCEEDIGLPKQFQHWMIQNYPVNEVMEIKGGDHMAMLSDPQKLCDCLSQISLKYA |
| CsSABP2-2 | MAEAKKQKHFVLVHGANHGAWCWYKVKPQLEAAGHRVTALDLAASGINVKKIEEVPTFYEYSEPLLEVLASLPAEEKVILVGHSFGGLSLALAADTFPHKISVAIFLTALMPDTKHQPSYVVDKLFNRVSSEEWLDTQFSVMDTSNPSHVSISFGHNFLTLKLYPLCSPQDVELGKMLARPGSLFQDDLSKANKFSNEGYGSVKRVYVVCDEDICIPKEFQHWMIQNNPVDQVMEIKGADHMPMLSKPQQLLHCLSQIAHKYA* |
| CsSABP2-3 | MKPTEKIKKMAEAKKRKHFVLVHGSNHGAWCWYKVKPQLEAAGYRVTALDLAATGINMKKIQDVHSFYEYNEPLLEFLASLSAGEKVVLVGHSAGGLSLALAADKFPHKISVAIFLTAFMPDTKHQPSYVVERFFEKIPSGEWLDTQFSVIDSSNPSRKTIFFGHNFLTLKLYQLSPPEDVELGKMLLRPGLVFVDELSKANKFSNEGYGSIKRVYVVCDEDICVPKQFQHWMIQNNPVDEVIEIKGVDHMPMLSKPHQVFDCLSHIAQKYA* |

**Table S2** Primers used in the study

| Primer name | Primer sequence（5′–3′） | Comments |
| --- | --- | --- |
| CsSABP2-1-f | TCCCCCGGGATGGAAGAAGTAGTAGGCAT | Construction of p35S:: SABP2-1 vector |
| CsSABP2-1-r | ACGCGTCGACTTATGCATACTTAAGAGAAA |  |
| CsSABP2-1^V18A^-f | TCCCCCGGGATGGAAGAAGTAGTAGGCAT | Construction of p35S:: SABP2-1^V18A^ vector |
| CsSABP2-1^V18A^-r | ACGCGTCGACTTATGCATACTTAAGAGAAA |  |
| CsSABP2-2-f | TCCCCCGGGATGGCAGAAGCCAAGAAACAGAA | Construction of p35S:: SABP2-2 vector |
| CsSABP2-2-r | ACGCGTCGACTTAAGCATACTTATGAGCAA |  |
| CsSABP2-3-f | TCCCCCGGGATGAAACCAACGGAGAAAAT | Construction of p35S:: SABP2-3 vector |
| CsSABP2-3-r | ACGCGTCGACTTAAGCATACTTTTGAGCAA |  |
| pPIC9K-CsSABP2-1-f | CGCTACGTAATGGAAGAAGTAGTAGGCATGGAAGAGAA | Construction of pAOX1:: SABP2-1 eukaryotic expression vector |
| pPIC9K-CsSABP2-1-r | ATAAGAATGCGGCCGCTTAGTGGTGGTGGTGGTGGTGTGCATACTTAAGAGAAATC |  |
| pPIC9K-CsSABP2-1^V18A^-f | CGCTACGTAATGGAAGAAGTAGTAGGCATGGAAGAGAA | Construction of pAOX1:: SABP2-1^V18A^ eukaryotic expression vector |
| pPIC9K-CsSABP2-1^V18A^-r | ATAAGAATGCGGCCGCTTAGTGGTGGTGGTGGTGGTGTGCATACTTAAGAGAAATC |  |
| pPIC9K-CsSABP2-2-f | CGCTACGTAATGGCAGAAGCCAAGAAACAGAAGCATTT | Construction of pAOX1:: SABP2-2 eukaryotic expression vector |
| pPIC9K-CsSABP2-2-r | ATAAGAATGCGGCCGCTTAGTGGTGGTGGTGGTGGTGAGCATACTTATGAGCAATT |  |
| pPIC9K-CsSABP2-3-f | CGCTACGTAATGAAACCAACGGAGAAAATTAAGAAAAT | Construction of pAOX1:: SABP2-3 eukaryotic expression vector |
| pPIC9K-CsSABP2-3-r | ATAAGAATGCGGCCGCTTAGTGGTGGTGGTGGTGGTGAGCATACTTTTGAGCAATG |  |
| 5´ AOX1 | GACTGGTTCCAATTGACAAGC | Recombinant colony PCR verification |
| 3´ AOX1 | GCAAATGGCATTCTGACATCC |  |
| OE—f  OE-CsSABP2-1-r | CTCCACTGACGTAAGGGATG  TGTCCAACCAGCTGTCGTCC | Universal primers upstream of gene expression analysis  Analysis of CsSABP2-1 expression |
| OE-CsSABP2-1^V18A^-r | TGTCCAACCAGCTGTCGTCC | Analysis of CsSABP2-1^V18A^expression |
| OE-CsSABP2-2-r | CATCACCTGATCAACGGGAT | Analysis of CsSABP2-2 expression |
| OE-CsSABP2-3-r | TTTCCCAGCTCCACATCCTC | Analysis of CsSABP2-3 expression |
| GADPH-f | GCTTTCCGTGTACCCACTGT | Quantitative Real-time PCR |
| GADPH-r  HLBas-f | CTCTGACTCCGCCTTGATGG  TCGAGCGCGTATGCAATACG | Quantitative Real-time PCR  Quantitative Real-time PCR |
| HLBas-r | GCGTTATCCCGTAGAAAAAGGTAG | Quantitative Real-time PCR |
| HLBp | AGACGGGTGAGTAACGCG | Quantitative Real-time PCR |
| RT-CsSABP2-1^V18A^-f  RT-CsSABP2-1^V18A^-r | TGGGAAAAGAGGACGACAGC TTGATCCTGGCCTCACCAAC | Quantitative Real-time PCR  Quantitative Real-time PCR |
| RT-CsSABP2-2-f | TGTGGATAAGCTTTTTAACCGGG | Quantitative Real-time PCR |
| RT-CsSABP2-2-r | ATCCTGGCCTCGCTAACATC | Quantitative Real-time PCR |
| RT-CsSABP2-3-f | TGTTGGAGTTTTTGGCCTCAC | Quantitative Real-time PCR |
| RT-CsSABP2-3-r | CCAAACTCAAGCCTCCAGCG | Quantitative Real-time PCR |
| RT-PR1-f | AAATGTGGGTGAATGAGAAAGC | Quantitative Real-time PCR |
| RT-PR1-r  RT-PR2-f  RT-PR2-r  RT-PR5-f  RT-PR5-r  RT-WRKY45-f  RT-WRKY45-r  RT-WRKY70-f  RT-WRKY70-r | ATTATTGTTGCACGTCACCTTG  TTCCACTGCCATCGAAACTG  GTAATCTTGTTTAAATGAGCCTCTTG  CACCATTGCCAATAACCCTAATG  GGGACAGTTACCGTTAAGATCAG  TGTACACACGAAGGGTGCAA  GCTCAAAGTTGTCAGTGGGC  GCTGCCAAGCAAGTAAGCAG  TTCGGTGATGTGGGCACTAC | Quantitative Real-time PCR  Quantitative Real-time PCR  Quantitative Real-time PCR  Quantitative Real-time PCR  Quantitative Real-time PCR  Quantitative Real-time PCR  Quantitative Real-time PCR  Quantitative Real-time PCR  Quantitative Real-time PCR |
| RT-NPR1-f  RT-NPR1-r  RT-NPR3-15-f  RT-NPR3-15-r  RT-NPR3-49-f  RT-NPR3-49-r  RT-NPR3-73-f  RT-NPR3-73-r  RT-NPR4-08-f  RT-NPR4-08-r | TCCAGAAGAGCGACTCCTGA  CACGAGGCCTCACAACTGAT  GCTTCATGTAGCTGCAAGGC  TCCCTGCTTTGTAGAGATTGCT  AAACAGTCGAGACGGGTGAC  TCTAGGAGGGACGCATCCAA  GAGGAGGAATCCGATGGCTG  ACCGTGCAAATGCCACTCTA  ACACCCTCTATGCAAGCCAAG  GCGCTTCCGGAGTACCAAAT | Quantitative Real-time PCR  Quantitative Real-time PCR  Quantitative Real-time PCR  Quantitative Real-time PCR  Quantitative Real-time PCR  Quantitative Real-time PCR  Quantitative Real-time PCR  Quantitative Real-time PCR  Quantitative Real-time PCR  Quantitative Real-time PCR |

**
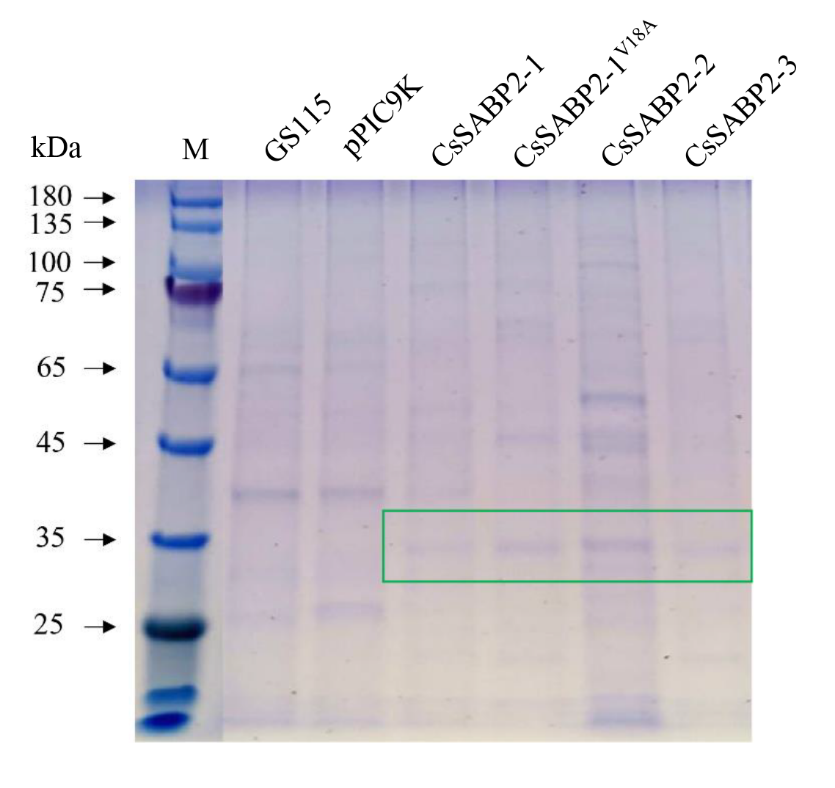
****Fig S1.** **SDS-PAGE analysis of purification of CsSABP2 protein from the yeast GS115 (*Saccharomyces cerevisiae*) transformed with *CsSABP2*.** M, Molecular weight markers; supernatant from the CsSABP2-1^V18A^ transformant; CsSABP2-2, supernatant from the CsSABP2-2 transformant; CsSABP2-3, supernatant from the CsSABP2-3 transformant, Within the box are the corresponding protein bands.

**
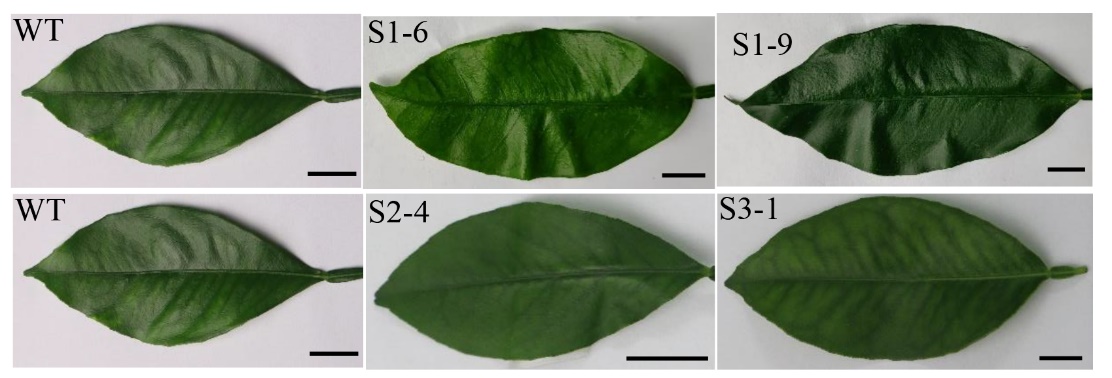
**

**Fig S2. Evaluation of Citrus Huanglongbing (HLB) tolerance in transgenic citrus plants**. HLB symptoms in the transgenic plants and WT plants after 7 MAI. WT, wild type; OE-#, transgenic plants. S1-#, *CsSABP2-1* transgenic plants, S2-#, *CsSABP2-2* transgenic plants, S3-#, *CsSABP2-3* transgenic plants.
